# *Clostridium butyricum* Prevents Dysbiosis and the Rise in Blood Pressure in Spontaneously Hypertensive Rats

**DOI:** 10.3390/ijms24054955

**Published:** 2023-03-04

**Authors:** Xianshu Luo, Zhuoyu Han, Qing Kong, Yuming Wang, Haijin Mou, Xuefeng Duan

**Affiliations:** College of Food Science and Engineering, Ocean University of China, Qingdao 266404, China

**Keywords:** *Clostridium butyricum*, butyrate, hypertension, short chain fatty acid, dysbiosis

## Abstract

Hypertension is accompanied by dysbiosis and a decrease in the relative abundance of short-chain fatty acid (SCFA)-producing bacteria. However, there is no report to examine the role of *C. butyricum* in blood pressure regulation. We hypothesized that a decrease in the relative abundance of SCFA-producing bacteria in the gut was the cause of spontaneously hypertensive rats (SHR)-induced hypertension. *C. butyricum* and captopril were used to treat adult SHR for six weeks. *C. butyricum* modulated SHR-induced dysbiosis and significantly reduced systolic blood pressure (SBP) in SHR (*p* < 0.01). A 16S rRNA analysis determined changes in the relative abundance of the mainly SCFA-producing bacteria *Akkermansia muciniphila*, *Lactobacillus amylovorus*, and *Agthobacter rectalis*, which increased significantly. Total SCFAs, and particularly butyrate concentrations, in the SHR cecum and plasma were reduced (*p* < 0.05), while *C. butyricum* prevented this effect. Likewise, we supplemented SHR with butyrate for six weeks. We analyzed the flora composition, cecum SCFA concentration, and inflammatory response. The results showed that butyrate prevented SHR-induced hypertension and inflammation, and the decline of cecum SCFA concentrations (*p* < 0.05). This research revealed that increasing cecum butyrate concentrations by probiotics, or direct butyrate supplementation, prevented the adverse effects of SHR on intestinal flora, vascular, and blood pressure.

## 1. Introduction

The intestinal flora’s potential role in influencing host health has attracted considerable attention in recent decades. Many metabolic diseases, inflammatory bowel diseases, and cardiovascular diseases have been reported to be linked to flora disorders [1,2,3]. In recent years, much seminal evidence has shown that abnormal intestinal flora is closely associated with changes in blood pressure in the host. Iñaki Robles-Vera and co-workers reported that fecal microbiota transplantation from adult spontaneously hypertensive rats (SHR), to adult Kyoto rats (WKY), resulted in a chronic rise in blood pressure (BP), vascular oxidative stress, and impaired endothelial function. Conversely, fecal microbiota transplantation from WKY to adult SHR induced a blood pressure reduction and improvement of endothelial dysfunction [4]. Mechanisms linking the gut microbiota to hypertension include dysbiosis, inflammation, gut permeability, and decreased production of short-chain fatty acids (SCFAs), particularly butyrate [5,6,7].

SCFAs are mainly produced through fermentation of indigestible carbohydrates by bacteria in the intestine [8], which can regulate body weight, energy metabolic balance, lipid metabolism, and other pathophysiological processes [9,10], including hypertension. SCFAs can be transported to the surface of the cell membrane, through mono-carboxylate transporter (MCT), to bind with G protein-coupled receptors (GPCRs), and modulate vascular tone and inflammation to regulate blood pressure through the interaction with GPCRs or inhibition of histone deacetylases [11,12]. Onyszkiewicz and co-workers demonstrated that butyrate could enter the circulation through the intestinal-vascular barrier, and act on GPR41/GPR43 to relax mesenteric arteries and decrease blood pressure [13]. In addition, treatment of Olfr78 knockout and non-knockout mice with propionate revealed that Olfr78 knockout mice showed lower blood pressure levels [14].

It has been reported that the probiotics *Bifidobacterium breve* CECT7263 and *Lactobacillus fermentum* CECT5716, could regulate the blood pressure of SHR through modulating short-chain fatty acid-producing genera in the intestine [15]. SHR is an established model of genetic hypertension characterized by elevated blood pressure, arterial remodeling, endothelial dysfunction, hyperlipidemia, vascular inflammation, and immune system dysregulation [16,17,18]. In addition, dysbiosis and a decrease in the relative abundance of SCFA-producing bacteria have been reported in SHR [19,20]. *Clostridium butyricum* (*C. butyricum*) is an anaerobic, gram-positive bacillus, known as a butyrate producer and a regulator of gut health [2]. It resides in the gastrointestinal tract and has a protective role against pathogenic bacteria and intestinal injury, via the modulation of gut microbial metabolites [21,22,23]. *C. butyricum* are capable of utilizing a range of carbohydrates, and produce several fermentation products, with butyrate as the major product. Butyrate plays a crucial role in promoting gut health and has been used in clinical practice to treat various chronic enteric diseases [24]. While probiotics and SCFAs have the potential to regulate blood pressure, we did not know if *C. butyrcium*, the main butyrate producer, could regulate blood pressure in SHR.

Based on the potential of probiotic and bacterial-derived SCFAs for blood pressure regulation, we hypothesized that *C. butyricum* could modulate intestinal flora, improve vascular inflammation, and lower blood pressure. We investigated the hypotensive effect of *C. butyricum* on SBP in SHR, using SHR animals as a model, and investigated the effects of SHR, WKY, and *C. butyricum* on intestinal immunity and vascular inflammation, a key component of SHR-induced hypertension. In addition, we analyzed the potential mechanistic link between dysbiosis and hypertension by 16S rRNA V1–V9 sequencing.

## 2. Results

### 2.1. C. butyricum and Butyrate Prevented SHR-Induced Hypertension

**Figure 1:**
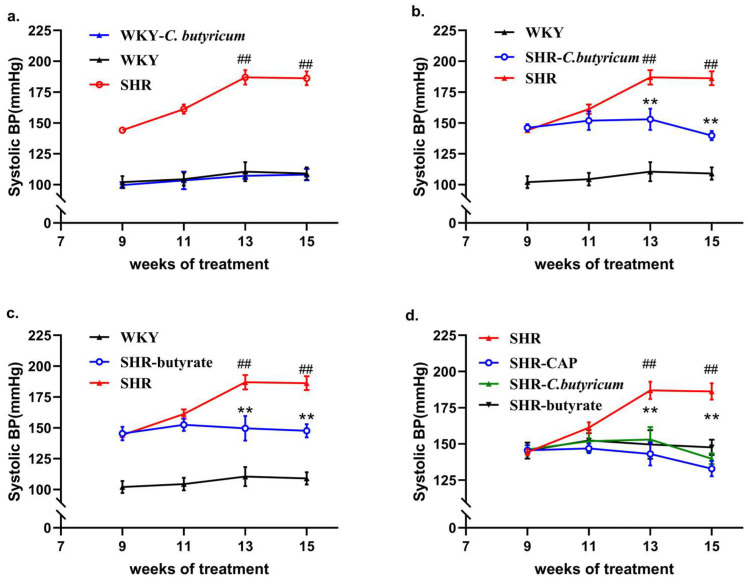
*C. butyricum* and butyrate prevented SHR-induced hypertension. (**a**) SHR rats exhibited significantly higher SBP compared to WKY, and *C. butyricum* had no significant effect on SBP of WKY (SHR, n = 8 per group, WKY, n = 6 per group, WKY-*C. butyricum*, n = 8 per group). (**b**) The SBP of SHR decreased significantly after 4 weeks of *C. butyricum* treatment (SHR-*C. butyricum*, n = 8 per group). (**c**) The SBP of SHR decreased significantly after 4 weeks of butyrate treatment (SHR-butyrate, n = 8 per group). (**d**) The SBP of SHR decreased significantly after 4 weeks of captopril treatment (SHR-CAP, n = 8 per group), *C. butyricum* and butyrate prevented elevated SBP in SHR at weeks 11–15, but their SBP was higher than that of rats in the captopril-treated group. Data are shown as the mean ± S.E.M., ** *p* < 0.01 for SHR vs. SHR-*C. butyricum* or SHR-butyrate. ## *p* < 0.01 for SHR vs. WKY.

In fed rats, the SBP of WKY remained around 100 mmHg, which was highly significantly different from that of SHR (*p* < 0.01). The SBP of SHR gradually increased between week 9 and week 13, and stabilized at 189 ± 7 mmHg by week 15. Given that multiple animal models of hypertension exhibit reduced SCFA-producing bacteria, we treated SHR with *C. butyricum* or butyrate (the main metabolite of *C. butyricum*). The SBP of SHR remained steadily lower throughout the treatment period of *C. butyricum* or butyrate. By week 15, the SBP of rats in the SHR-Cb group was 50 mmHg lower than that of the rats in the SHR group (Figure 1b,c). In contrast, there was no significant difference in SBP between rats in the WKY-*C. butyricum* group compared to those in the WKY group (Figure 1a). This suggests that *C. butyricum* was able to prevent elevated SBP in SHR but did not affect normotensive rats. Captopril lowers blood pressure in hypertension. The results of the repeated measures ANOVA showed that *C. butyricum* treatment significantly reduced the SBP in SHR after two weeks (Tables S1–S3). In addition, to clarify the effect of *C. butyricum* in preventing elevated blood pressure, we treated SHR with captopril for 6 weeks. The results showed that the SBP of SHR was significantly reduced after 2 weeks of treatment (Tables S1–S3), but *C. butyricum* or butyrate had a significant effect in preventing elevated SBP after 4 to 6 weeks of treatment (Figure 1d).

### 2.2. C. butyricum and Butyrate Altered the Colonic Microbiota Composition

**Figure 2:**
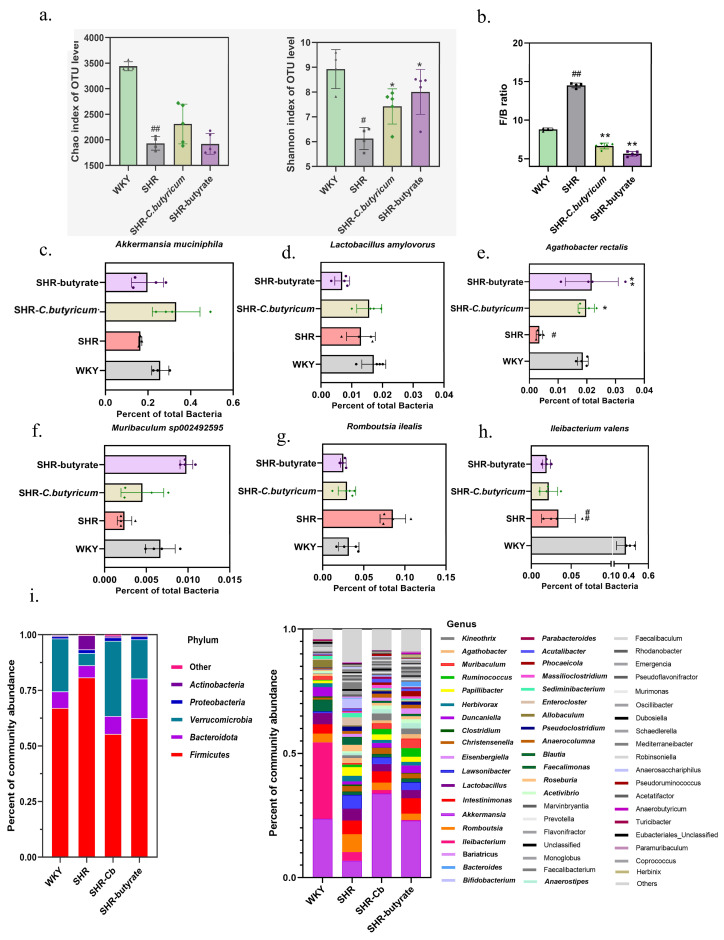
*C. butyricum* and butyrate alter the composition of the intestinal microbiota. (**a**) 16S rDNA V3–V4 sequencing to evaluate Chao richness and Shannon diversity. (**b**) The *Firmicutes*/*Bacteroidetes* ratio was calculated as a biomarker of intestinal flora dysbiosis. (**c**–**h**) Relative abundance of *Akkermansia muciniphila*, *Lactobacillus amylovores*, *Agthobacter rectails*, *Muribaculum* sp002492595, *Romboutsia ilealis*, and *lleibacterium valens*. (**i**) The colors of community abundance percentage plots represent the relative percentage of microbial families assigned to each group. Data are shown as the mean ± S.E.M. n = 4 (WKY, SHR) or 5 (all other groups) for a and c, n = 4 for all groups in c to h, * *p* < 0.05, ** *p* < 0.01 for SHR vs. SHR-*C. butyricum* or SHR-butyrate. # *p* < 0.05, ## *p* < 0.01 for SHR vs. WKY.

**Figure 3:**
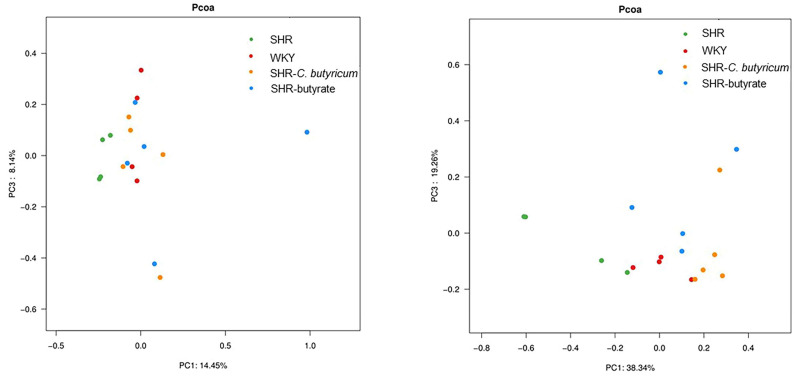
Beta diversity of gut microbes analyzed by PCoA. PCoA used unweighted UniFrac analysis and weighted UniFrac analysis to calculate the distances between the six groups in the colonic stool samples (SHR and WKY, n = 6 per group, SHR-*C. butyricum* and SHR-butyrate, n = 8 per group).

To investigate the effect of *C. butyricum* and butyrate intervention on gut microbiology, we analyzed the full-length 16S rRNA sequencing of the colon contents. The richness and diversity of the intestinal flora were lower in SHR than in WKY (*p* < 0.05). *C. butyricum* increased the richness and diversity of the colonic flora by increasing the values of Chao and Shannon at the OUT level (Figure 2a), while butyrate did not alter the abundance of colonic flora. Similarly, *C. butyricum* and butyrate showed a clear separation from SHR alone in both unweighted and weighted UniFrac principal coordinate analyses (Figure 3). *Firmicutes*, *Bacteroidetes*, *Actinobacteria*, *Proteobacteria*, *Fusobacteria*, and *Verrucomicrobia* are the main phyla in the gut, with *Firmicutes* and *Bacteroidetes* accounting for 90% of the intestinal flora [25,26]. As shown in Figure 2c, *Firmicutes* were significantly increased in the SHR colon compared to WKY (WKY: 67%, SHR: 80%), while the relative abundance of *Proteobacteria* in the SHR colon was 6% higher than that of WKY. *C. butyricum* and butyrate significantly increased the relative abundance of *Verrucomicrobia* and *Bacteroidetes* in the SHR colon (*p* < 0.01). This indicates that *C. butyricum* and butyric acid altered the intestinal microbial community of SHR. On the genus level, the relative abundance of *Akkermansia* in the colon of SHR was significantly increased by *C. butyricum* and butyrate (SHR: 4%, SHR-*C. butyricum*: 33%, SHR-butyrate: 18%) (Figure 2c).

The *Firmicutes*/*Bacteroides* ratio is a marker of intestinal health. Some diseases such as inflammatory bowel disease and excessive obesity, including hypertension, have been reported to have significantly higher *Firmicutes*/*Bacteroides* ratios, and regulation of the ratio is effective in treating the disease [27,28]. We analyzed *Firmicutes*/*Bacteroides* in all groups of rats, and we demonstrated that *Firmicutes*/*Bacteroides* were significantly higher in SHR than WKY (*p* < 0.01). Treatment with *C. butyricum* and butyrate prevented *Firmicutes*/*Bacteroides* from being elevated (*p* < 0.01) (Figure 2b).

In addition, we analyzed changes in colonic microorganisms on the species level of SHR after 6 weeks of *C. butyricum* and butyrate treatment. Consistent with previous studies, short-chain fatty acid-producing bacteria were decreased in the SHR gut. *Akkermansia muciniphila*, *Lactobacillus amylovorus*, *Muribaculum* sp002492595, *Agthobacter rectalis*, *Romboutsia ilealis*, and *lleibacterium valens* had their relative abundance altered by *C. butyricum* and butyrate (Figure 2c–h). We demonstrated that *C. butyricum* and butyrate prevented the decrease in the relative abundance of *Akkermansia muciniphila*, *Muribaculum* sp002492595, and *Agthobacter rectalis* in the colonic contents caused by SHR (Figure 2c–e). *C. butyricum* and butyrate significantly increased *Akkermansia muciniphila* (SHR-*C. butyricum*: 34%, SHR-butyrate: 23%) (Figure 2c), interestingly, Roshanravan N. and co-workers reported that supplementation with butyrate promoted *Akkermansia muciniphila* levels in the intestine, improved vascular inflammation and oxidative stress, and lowered blood pressure [29,30], which is consistent with the results of our study. Both *Lactobacillus amylovorus* and *lleibacterium valens* have been reported to reduce obesity [31,32]. The results showed that colonic *Lactobacillus amylovorus* increased after 6 weeks of *C. butyricum* treatment, but that butyrate did not alter their relative abundance in the colon (Figure 2d), while treatment with *C. butyricum* and butyrate did not alter the colonic levels of *lleibacterium valens* (Figure 2h). In addition, treatment with both *C. butyricum* and butyrate reduced the levels of the SHR colonic pathogen *Romboutsia ilealis* (Figure 2g).

### 2.3. Inflammation of the Colon and Vascular Improved by C. butyricum and Butyrate

**Figure 4:**
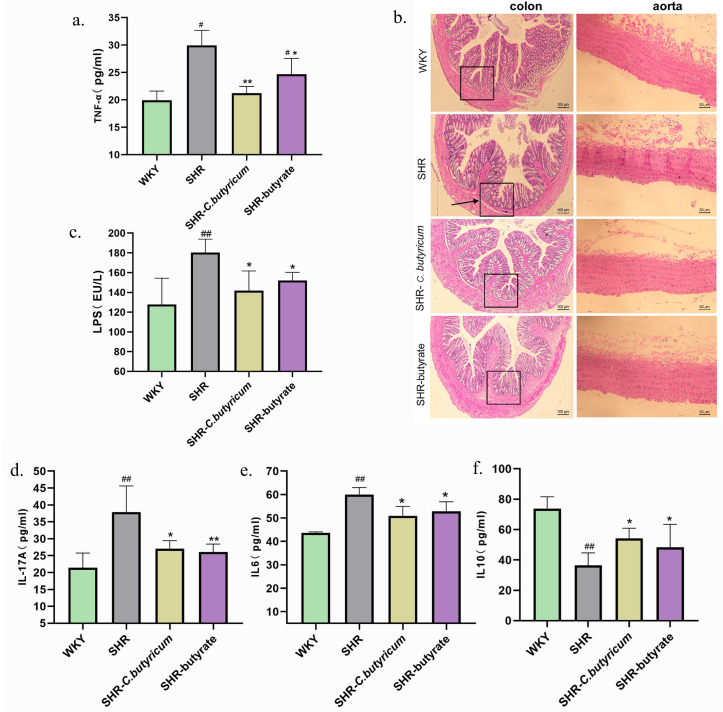
Inflammation of SHR is reduced by *C. butyricum* and butyrate. (**a**,**c**–**f**) *C. butyricum* and butyrate regulated cytokine levels (**a**, TNF-α; **c**, LPS; **d**, IL-17A; **e**, IL-6; **f**, IL-10; n = 3 per group). (**b**) H&E staining of the colon and aorta. Data are shown as the mean ± S.E.M., * *p* < 0.05, ** *p* < 0.01 for SHR vs. SHR-*C. butyricum* or SHR-butyrate. # *p* < 0.05, ## *p* < 0.01 for SHR vs. WKY.

We examined the effects of SHR on the colon and vascular system with and without *C. butyricum* and butyrate. Figure 4b shows that SHR caused severe intestinal mucosal detachment in the colon, goblet cells in the colon were reduced, and there was a proliferation of inflammatory fibrous tissue; all of these were prevented by treatment with *C. butyricum* and butyrate. In addition, we observed that SHR caused endothelial cell damage, proliferation, and shedding of outer membrane cells in the aorta. Treatment with *C. butyricum* and butyrate improved the vascular damage caused by SHR (Figure 4b). Since SHR caused inflammation in the colon and vascular system, we next examined serum levels of inflammatory factors. ELISA demonstrated that *C. butyricum* and butyrate decreased tumor necrosis factor-α (TNF-α), interleukin-6 (IL-6), interleukin-17A (IL-17A), and lipopolysaccharide (LPS), and increased the inhibitory inflammatory factor interleukin-10 (IL-10) in the SHR circulation (Figure 4a,c–f). This suggests that the regulation of the SBP in SHR by *C. butyricum* and butyrate possibly correlates with reduced levels of inflammation.

### 2.4. SHR Reduced Cecal and Plasma SCFAs, Which Were Prevented by C. butyricum and Butyrate

**Figure 5:**
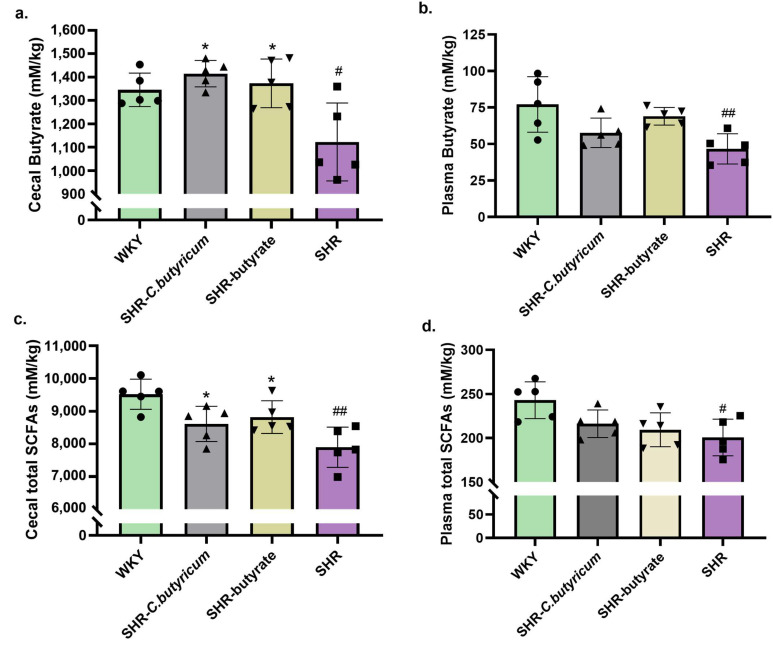
SHR reduced cecal and plasma SCFAs concentration, which was prevented by *C. butyricum* and butyrate. (**a**,**c**) Cecal and plasma total SCFAs concentration were significantly increased by *C. butyricum* and butyrate (n = 5 per group). (**b**,**d**) The reduction in SCFAs concentration in SHR cecal and plasma was prevented by *C. butyricum* and butyrate (n = 5 per group). Data are shown as the mean ± S.E.M., * *p* < 0.05 for SHR vs. SHR-*C. butyricum* or SHR-butyrate. # *p* < 0.05, ## *p* < 0.01 for SHR vs. WKY.

**Figure 6:**
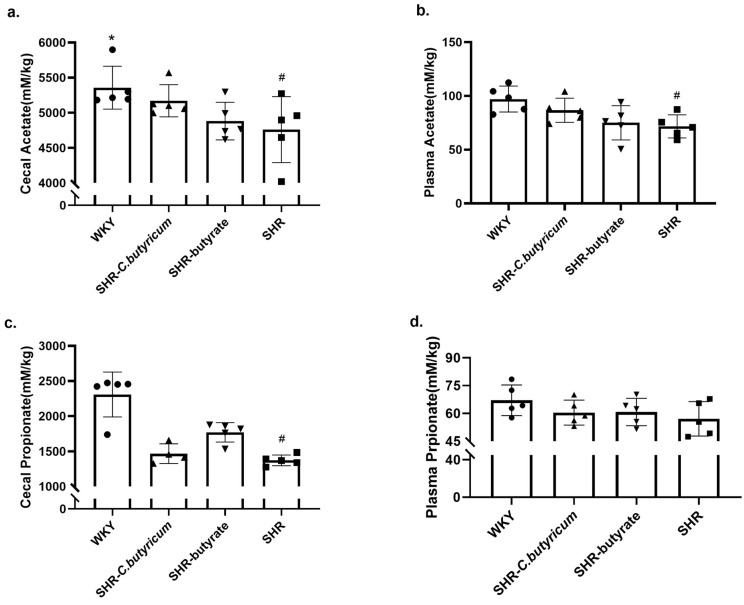
SHR reduced cecal and plasma SCFAs concentration, which was prevented by *C. butyricum* and butyrate. (**a**,**b**) Cecal and plasma acetate concentration was increased by *C. butyricum* and butyrate (n = 5 per group). (**c**,**d**) the reduction in propionate concentration in SHR cecal and plasma was prevented by *C. butyricum* and butyrate (n = 5 per group). Data are shown as the mean ± S.E.M., * *p* < 0.05 for SHR vs. SHR-*C. butyricum* or SHR-butyrate. # *p* < 0.05 for SHR vs. WKY.

To clarify the effect of dysbiosis on SHR, we next tested whether the SHR-induced changes to the microbiota led to changes in microbial metabolites that may contribute to the adverse effects of SHR on the gut and blood pressure. Microbiota analysis showed that some short-chain fatty acid producers were modified by SHR, which was prevented by *C. butyricum* and butyrate (Figure 2d–h). Therefore, we measured the SCFAs concentrations in colonic contents and plasma. SHR caused a significant decrease in colonic and plasma total SCFAs, particularly butyrate concentrations (Figure 5). *C. butyricum* and butyrate significantly prevented the SHR-induced decrease in colonic butyrate, acetate, and propionate concentrations, but the effects on acetate and propionate were not significant (Figure 5c and Figure 6). SHR caused a decrease in total SCFAs in plasma, but we found that SHR did not change the concentration of propionate in plasma (Figure 5b and Figure 6d).

### 2.5. Relative Expression Levels of SCFA Transporters in the Proximal Colon of WKY and SHR

**Figure 7:**
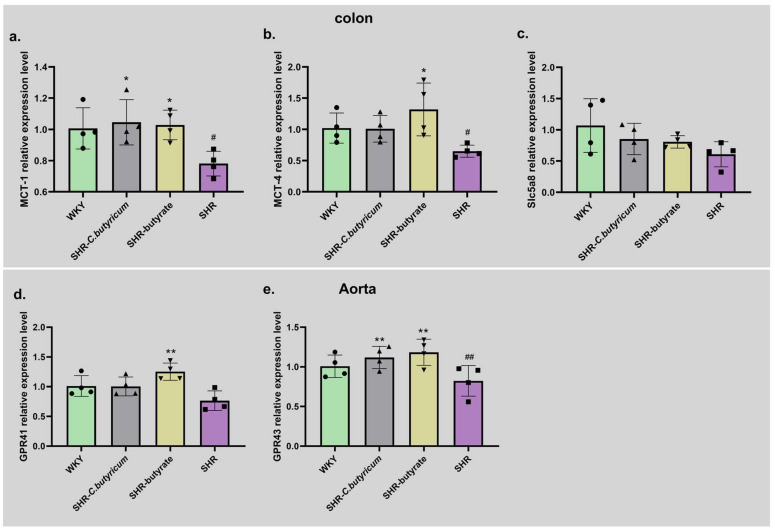
The relative expression levels of SCFAs transporters and SCFA-sensing receptors were altered by *C. butyricum* or butyrate. (**a**–**c**) *C. butyricum* or butyrate prevented the decrease in the relative expression of colonic SCFAs transporter proteins MCT1, MCT4, and Slc5a8 caused by SHR. (**d**,**e**) SHR significantly decreased the relative expression levels of the aortic SCFA-sensing receptors GPR41, GPR43 mRNA (GPR41, *p* = 0.055 for WKY vs. SHR; GPR43, *p* < 0.001 for WKY vs. SHR). The relative expression levels of GPR41 and GPR43 were significantly increased after treatment with *C. butyricum* or butyrate. Data are shown as the mean ± S.E.M., * *p* < 0.05, ** *p* < 0.01 for SHR vs. SHR-*C. butyricum* or SHR-butyrate. # *p* < 0.05, ## *p* < 0.01 for SHR vs. WKY (n = 4 per group).

We tested the relative expression levels of three monocarboxylate transporter proteins involved in transporting butyrate and other SCFAs into and through the intestinal epithelium [11,33]. Figure 7a,b shows that the relative expression levels of MCT1 and MCT4 were significantly reduced in the colon of SHR compared to WKY (*p* < 0.05), which was enhanced by treatment with *C. butyricum* or butyrate. Similarly, the relative expression level of Slc5a8 was also enhanced by *C. butyricum* or butyrate, but it was not significant (n = 4 per group, *p* = 0.229 for SHR vs. SHR-*C. butyricum*, *p* = 0.324 for SHR vs. SHR-butyrate). This result suggested that the decrease in circulating total SCFAs and butyrate concentration may be due to a decrease in SCFAs transport proteins caused by SHR.

### 2.6. Reduced Expression of SCFA-Sensing Receptors in the SHR Vascular

To evaluate the direct effects of SCFAs on the vasculature, we examined the relative expression levels of SCFA-sensing receptors in the aorta. Most notable among the SCFA targets is the mammalian G protein-coupled receptor pair of GPR41 and GPR43, that can be expressed in blood vessels [34]. We observed reduced relative expression levels of GPR41 and GPR43 in the SHR aorta (GPR41, *p* = 0.051; GPR43, *p* = 0.004 for WKY vs. SHR). After 6 weeks of treatment, the mRNA expression levels of GPR41 and GPR43 in the SHR- *C. butyricum* group and SHR-butyrate group were upregulated compared with those in the SHR group, meanwhile, compared with the *C. butyricum* group, the results showed that butyrate treatment group had a more significant regulatory effect (*p* < 0.01). Therefore, the mechanism for sensing SCFAs appears to be partially compromised in the SHR aorta, but was ameliorated by treatment with *C. butyricum* or butyrate.

### 2.7. SHR Increased Th17/Treg in the Spleen and Aorta and C. butyricum Restored Th17/Treg

**Figure 8:**
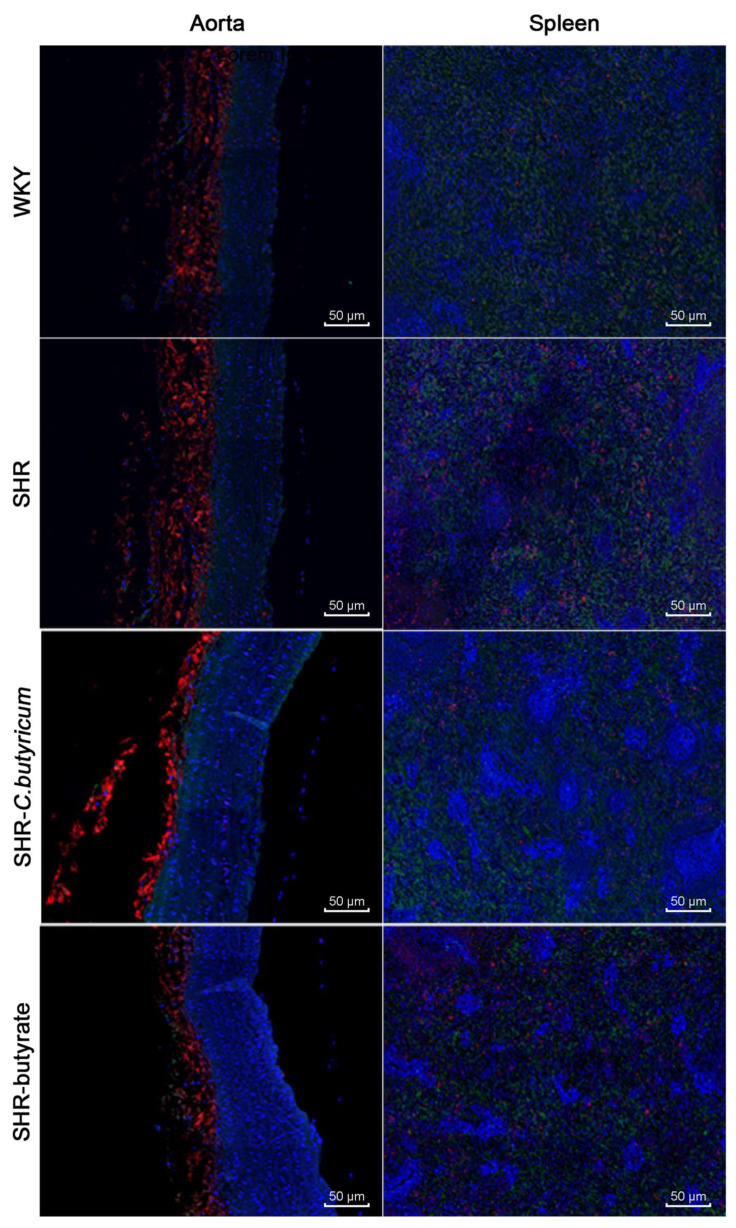
SHR increased Th17 and decreased Treg in the aorta and spleen, which could be prevented by *C. butyricum* or butyrate treatment. Th17 staining is shown in red, Treg staining is shown in green, and nuclear DAPI staining is shown in blue.

The host inflammation is associated with a balance between the pro-inflammatory and anti-inflammatory actions of regulatory T cells. The Th17/Treg balance has been shown to normalize endotoxemia, prevent endothelium-dependent diastolic damage to acetylcholine, and reduce blood pressure in spontaneously hypertensive rats [30]. Therefore, we examined the distribution of Th17 and Treg in the aorta and spleen. Figure 8 shows that SHR leads to Th17 increases and Treg decreases in the spleen and aorta. These were ameliorated by treatment with *C. butyricum* or butyrate.

## 3. Discussion

In recent years, much seminal evidence has demonstrated for the first time that abnormal gut flora is closely associated with changes in blood pressure in the host [35]. Both animal models of hypertension and human hypertension are accompanied by dysbiosis [36]. Through the study of the effect of *C. butyricum* on the intestinal flora, we have demonstrated that C. *butyricum* could regulate the intestinal flora and increase the relative abundance of short-chain fatty acid-producing bacteria [37]. We hypothesized that a reduction in SCFAs was responsible for SHR hypertension.

Probiotics such as *Bifidobacterium* breve CECT7263 and *Lactobacillus fermentum* CECT5716, have been shown to restore SHR-induced dysbiosis and reduce SBP [15]. These probiotics were found to ferment dietary fiber in the gut to produce SCFAs, which protect against the vascular oxidative stress and endothelial dysfunction caused by hypertension. Several studies have shown that the relative abundance of many SCFA-producing bacteria is reduced in animal models of hypertension [19,20,38]. Bhanu P. Ganesh and co-workers have demonstrated that *C. butyricum* reduced the effect of hypertension in a model of obstructive sleep apnea on altered microbiota, and increased the relative abundance of many SCFA-producing genera such as *Parabacteroides*, *Roseburia*, *Clostridium*, *Bifidobacterium*, *Ruminococcus*, and *Blauti* [39]. We suggested that *C. butyricum* and butyrate reduced the effect of SHR on altering the microbiota. Unweighted and weighted UniFrac principal coordinate analyses were used to show that the flora of SHR and WKY were significantly separated, altered by *C. butyricum* and butyrate. Chao and Shannon showed that *C. butyricum* and butyrate restored the reduction in flora diversity and abundance caused by SHR. In addition, treatment with *C. butyricum* and butyrate significantly reduced the *Firmicutes*/*Bacteroides* ratio (*p* < 0.01). We concluded that SHR induced dysbiosis and elevation of *Firmicutes*/*Bacteroides*, which was prevented by *C. butyricum* or butyrate. On a species-level analysis of all rat colon flora, we found that *C. butyricum* and butyrate prevented SHR-induced decreases in colonic *Akkermansia muciniphila*, *Muribaculum* sp002492595, and *Agthobacter rectalis* levels, which is consistent with previous findings. *Akkermansia muciniphila* is a native bacterium in the gut that ferments carbohydrates in the intestine to produce acetate and propionate, reducing metabolic disorders and improving low levels of inflammation [40]. Levels of intestinal *Akkermansia muciniphila* are negatively correlated with diabetes, obesity, and other metabolic syndromes [41]. Interestingly, previous studies have reported that supplementation with butyrate promotes intestinal levels of *Akkermansia muciniphila*, reduces vascular inflammation and oxidative stress, and lowers blood pressure [30]. Thus, *Akkermansia muciniphila* might play a significant role in reducing the SBP in SHR. In conclusion, *C. butyricum* was demonstrated to modulate the intestinal flora of SHR, reduce the *Firmicutes*/*Bacteroides* ratio, and prevent the SHR-induced reduction of short-chain fatty acid-producing species, thereby potentially maintaining colonic butyrate levels.

SCFAs, a major source of energy for epithelial cells, have many beneficial effects, including maintaining the integrity of the intestinal barrier [42], reducing mucosal inflammation, and improving intestinal health [43,44]. We found that SHR caused a significant reduction in colon and plasma SCFAs, particularly butyrate concentrations. *C. butyricum* and butyrate prevented the SHR-induced decrease in colon butyrate concentrations, but had no significant effect on acetate and propionate. SHR caused a decrease in the total SCFAs of plasma, but it did not modify plasma propionate concentrations. SCFAs could affect the host by activating the G protein-coupled receptor (GPCR) or by inhibiting histone deacetylases, to stabilize the intestinal epithelial barrier, regulate cytokine secretion, alter the T lymphocyte population, increase the protective mucus layer, and regulate antibody secretion [45,46,47,48]. If SCFAs entered the circulatory system, it would also affect tissues and organs outside the intestinal tract [49]. We observed that SHR induced a reduction in plasma concentrations of SCFAs, which was not significantly improved by *C. butyricum* and butyrate; so we examined the relative expression of SCFAs transporters in the proximal colon and SCFA-sensing receptors. We demonstrated that SHR caused a reduction in colonic MCT1 and MCT4 expression, which was prevented by *C. butyricum* or butyrate. Similarly, the reduction in the SCFA-sensing receptors GPR-41 and GPR-43 mRNA expression in the aorta of SHR, was ameliorated by *C. butyricum* or butyrate.

Treatment with *C. butyricum* and butyrate increased the concentration of intestinal SCFAs, while having no significant effect on plasma SCFAs. Therefore, we examined the possibility of colonic and aortic effects. Our findings in this study showed that the number of mucus-producing goblet cells of the colon was reduced, and there was intestinal mucosal detachment in SHR, and this could be prevented by *C. butyricum* and butyrate treatment. Similarly, Santisteban and co-workers reported a reduction in goblet cells in the SHR colon [50]. Similarly, *C. butyricum* and butyrate improved SHR-induced vascular injury. LPS could increase the intestinal permeability, and an increased intestinal permeability would allow more LPS to enter the circulation and exacerbate the inflammatory state [51]. Iñaki Robles-Vera and co-workers reported that LPS levels in SHR serum were significantly higher than WKY, and after treatment with the probiotic *Bifidobacterium* bifidum and SCFAs, blood pressure and LPS were normalized in the treated group of rats. They hypothesized that a reduction in LPS in rat serum was a key factor in the modulatory effect of probiotics on hypertension [15]. Our results have shown that treatment with *C. butyricum* and butyrate reduced LPS levels in serum of SHR.

In addition, TNF-α is a pro-inflammatory factor produced by macrophages, and plays an important pathogenic role in inflammatory bowel disease. The downregulation of TNF-α can significantly downregulate the inflammation of Crohn’s disease, and the content is positively correlated with inflammation [52]. IL-10 is an anti-inflammatory cytokine produced by immature T cells. Previous studies have shown that 17 strains of clostridium from healthy human microbiota can induce the production of IL-10, thus inhibiting colitis. The probiotic *C. Butyricum* can promote the production of IL-10 by T cells and thus prevent the occurrence of colitis through an IL-10-dependent mechanism [53]. IL-6 and IL-17A are pro-inflammatory factors produced by Th17 cells. IL-6 is an important cytokine in the process of immune inflammatory reaction, its abnormal content will damage the vascular endothelium and lead to an increase in blood pressure in hypertensive patients [54]. We observed that serum levels of the inflammatory factors TNF-α, IL-6, and IL-17A were increased by SHR and decreased by *C. butyricum* or butyrate. Conversely, IL-10 was increased by *C. butyricum* or butyrate. These findings suggest that *C. butyricum* and butyrate ameliorate vascular inflammation and modulate the SBP of SHR, which may be linked to the modulation of inflammatory factor levels. The balance between Th17 and Treg is critical for maintaining immune homeostasis, with the over-activation of Th17 cells exacerbating intestinal inflammation, while the lack of Treg in intestine-associated lymphoid tissue, or its inability to circulate naturally to sites of inflammation, has been shown to cause an immune response in commensal flora, and to induce colitis [55]. In this study, to analyze the effect of SHR on Th17 and Treg, we performed immunofluorescence staining of the spleen and aorta. We concluded that SHR induced an increase in splenic and aortic Th17 and a decrease in Treg, which improved through treatment with *C. butyricum* or butyrate.

**Figure 9:**
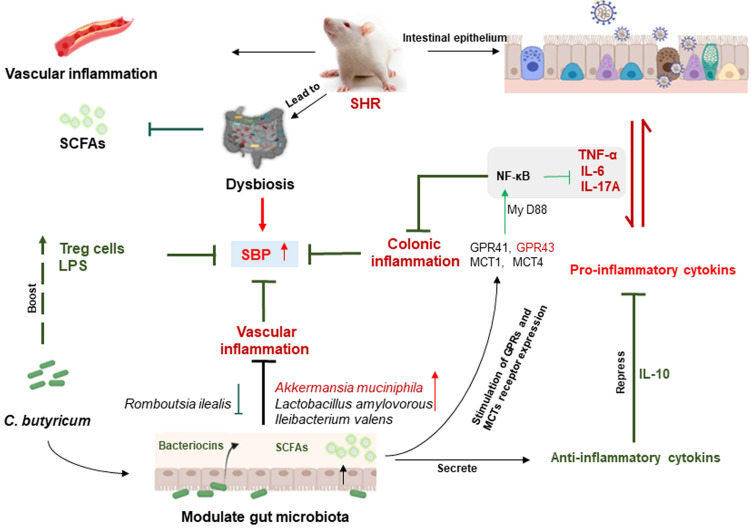
The mechanism by which *C. butyricum* reduces the SBP of SHR.

We have shown that (1) SHR caused a decrease in colonic SCFAs, especially butyrate concentrations, and increased intestinal and vascular inflammation, and hypertension. (2) in SHR rats, the probiotic *C. butyricum,* and SCFA butyrate, increased total colonic SCFAs and butyrate concentrations, reduced dysbiosis and colonic injury, and prevented vascular inflammation and hypertension. (3) *C. butyricum* and butyrate regulated the expression level of the major SCFAs transporters MCT1 and MCT4, and the receptors GPR41 and GPR43, in SHR rats. They may effectively regulate the colonic inflammation of SHR by regulating the concentration of SCFAs. (4) *C. butyricum* and butyrate modulated the signal pathway, to elevate the anti-inflammatory level by reducing the expression level of pro-inflammatory factor TNF-α, IL-6 and IL-17A, and improving the expression level of anti-inflammatory factor Il-10. (5) *C. butyricum* and butyrate decreased the level of intestinal LPS, and then ameliorated the intestinal barrier dysfunction caused by SHR. These findings demonstrate the critical role of impaired butyrate production in the development of SHR-induced hypertension, and suggest that treatment targeting increased *C. butyricum* and microbial butyrate production may prove effective in treating hypertension (Figure 9).

## 4. Materials and Methods

### 4.1. C. butyricum Cultivation

*C. butyricum* CGMCC 1.5205 (*C. butyricum*) was preserved at the China General Microbiological Culture Collection Center (CGMCC). A thermo-static incubator (DRP-9052, Senxin, Shanghai, China) was used to cultivate *C. butyricum* in reinforced *Clostridium* medium for 24 h at 37 °C. 16S rDNA sequencing (27F: AGAGTTTGATCCTGGCTCAG, 1492R: TACGGCTACCTTGTTACGACTT) was performed on the broth of *C. butyricum*, and the sequencing results were compared on the NCBI website (https://www.ncbi.nlm.nih.gov (accessed on 25 January 2022)) to confirm that the strain cultivated was *C. butyricum*. The *C. butyricum* fermentation broth was centrifuged at 10,000× *g* rpm for 10 min and 20% trehalose was added to the bacterial pellets as a protective agent before freeze-drying. The freeze-dried bacteria were counted using the blood counting chamber, with a result of 10^10^ cfu/mL. The freeze-dried powder was stored until use.

### 4.2. Experimental Animals

All studies in animals should be conducted in accordance with the National Institutes of Health (NIH) Guide for the Care and Use of Laboratory Animals, or the equivalent. All experimental procedures were approved by the Animal Ethics Committee of Ocean University of China (Approved protocol no: SPXY2017050402).

Thirty-two male, 5-week-old spontaneously hypertensive rats (SHR) and 12 Kyoto rats (WKY) were purchased from Beijing Viton Lever Laboratory Animal Technology Co. (Beijing, China). After 4 weeks of acclimatization feeding, 32 SHR rats were randomly divided into 4 groups (n = 8) and 12 WKY rats were randomly divided into 2 groups (n = 6), according to body weight and blood pressure. The WKY group (gavaged with 3 mL of sterilized saline NaCl 0.8%), WKY *C. butyricum* group (WKY-Cb) (3 mL of *C. butyricum* freeze-dried powder dissolved in sterilized normal saline (10^8^ CFU/mL) was administered to the rats), SHR group (gavaged with sterilized saline NaCl 0.8%), SHR *Clostridium butyricum* (SHR-*C. butyricum*) (3 mL of *C. butyricum* freeze-dried powder dissolved in sterilized normal saline (10^8^ CFU/mL) was administered to the rats), SHR butyrate group (0.5 mg/kg), and SHR captopril group (SHR-CAP) (captopril 10 mg/kg, Changzhou Pharmaceutical factory). All groups were gavaged once a day. Rats were housed in individual ventilated cages, in a pathogen-free animal facility under temperature (20–22 °C) and humidity (50–60%) control, and with a pre-set light-dark cycle (12 h:12 h). During the experimental period, the rats had free access to tap water and feed. All rats were executed after 6 weeks, and the intestinal contents, spleen, aorta, and serum were collected and stored at −80 °C until use.

### 4.3. Blood Pressure Measurements

In a consistent environment, to reduce disturbances for blood pressure measurement of the rats, 44 rats were acclimatized and fed for 4 weeks before being prepared for grouping.

The SBP was measured in unanesthetized rats using the CODA volume-pressure relationship tail-cuff system (Kent Scientific Corporation, Muscatine, IA, USA). Blood pressure was measured at regular intervals, every 2 weeks, during the treatment period, consecutive measurements were taken for each one, and the blood pressure data were averaged for each measurement.

### 4.4. Quantitative Real-Time PCR of Colonic Tissue

Gene expression in colonic tissue was measured by quantitative real-time PCR with SYBR Green. Quantitative real-time PCR was performed on a Thermo Lifetech ABI QuantStudio 3 from Applied Biosystems (Applied Biosystems, Thermo Fisher, Waltham, MA, USA). All amplification reactions were carried out in 96-well optical-grade PCR plates in triplicate (Applied Biosystems, Thermo Fisher, USA), each with 20 μL, sealed with optical sealing tape (Ruibiotech, Qingdao, China). The primers were designed with the GenBank database or DNAMAN for Windows, and synthesized commercially by Ruibiotech. All primers used are shown in Table S4.

### 4.5. DNA Extraction and 16S rRNA Gene Sequencing

The TIANGEN Bacterial Genomic DNA Extraction Kit (DP302-02, TIANGEN, Beijing, China) was used to extract colonic fecal genomic DNA. Colonic feces were collected under aseptic conditions and stored at −80 °C until subsequent analysis. Genomic DNA from the colonic fecal samples was extracted using the TIANGEN Fecal Genomic DNA Extraction Kit (DP328-02, TIANGEN, Beijing, China) according to the manufacturer’s instructions. A NanoDrop spectrophotometer ND 3.0 1000 (NanoDrop Technologies, Wilmington, DE, USA) was used to quantify the concentration of extracted DNA. The purity of the extracted DNA was checked by 1% agarose gel electrophoresis.

PacBio 16S rRNA V1–V9, sequenced and analyzed on the Pacbio Sequel II sequencing platform, was performed by Biozeron (Lingen, Shanghai, China). The recovered purified PCR products were detected and quantified by a QuantiFluor™-ST Blue Fluorescence Quantification System (Promega, Madison, WI, USA), then mixed in the appropriate proportions according to the sequencing volume required for each sample, and analyzed in PacBio libraries.

### 4.6. Bioinformatic Analysis of Sequencing Data

The raw data from PacBio were processed using the SMRT analysis software, version 9.0, to obtain demultiplexed circular consensus sequence (CCS) reads. OUT clustering was performed using UPARSE (version 7.1), based on 98.65% similarity (http://drive5.com/uparse/ (accessed on 12 February 2022)), and chimeric sequences were identified and removed by UCHIME. The phylogeny of each 16S rRNA gene sequence was analyzed by the RDP classifier (http://rdp.cme.msu.edu/ (accessed on 18 February 2022)) against the Silva (SSU132) 16S rRNA database, multiple diversity index analysis based on OUT data, and statistical analysis of community structure.

### 4.7. Enzyme-Linked Immunosorbent Assay (ELISA)

The levels of IL-6, TNF-α, IL-10, IL-17A, and LPS in serum were measured using ELISA kits (Lianke, Hangzhou, China). All procedures were performed according to the steps of the kits’ instructions. Plasma was centrifuged at 3500 rpm for 15 min and three duplicate samples were prepared for each group. An enzyme marker was used to collect the fluorescence intensity. After removing background and normalization, the concentration of each cytokine in the sample was calculated from the standard curve.

### 4.8. Histological Analysis of Colon, Spleen and Aortic Tissue

A suitably sized colonic, splenic, and aortic ring was fixed in 10% (*v*/*v*%) neutral buffered formalin, then washed in running water, dehydrated in alcohol, cleaned in xylene, and treated with paraffin. Thin tissue sections (3–5 μm) were then rehydrated and stained with hematoxylin and eosin (H&E) [56]. After staining, the dried slides were photographed and preserved using a microscope (BX41, Olympus Corporation, Tokyo, Japan) to observe the state of the transverse colon, spleen, and aorta.

### 4.9. Immunofluorescence Staining of Aorta and Spleen

Immunofluorescence assays were performed by Servicebio (Wuhan, China). Aortic slides were dewaxed, hydrated, and then subjected to microwave antigen repair in ethylenediaminetetraacetic acid buffer (pH 9.0). After serum closure (4% goat serum for 40 min), sections were incubated with the following primary antibody combinations: anti-CD4 and anti-IL-10, anti-CD4, and anti-IL-17, diluted overnight at 4 °C at 1:100. After incubation with the primary antibodies, the slides were incubated with the secondary antibodies for 50 min, protected from light. In addition, the slides were stained with a 1:200 dilution of 4′-6-diamidino-2-phenylindole (DAPI) solution in the dark for 10 min. Finally, the stained cells were observed by fluorescence microscopy (BX41, Olympus Corporation, Tokyo, Japan) and images were collected.

### 4.10. Statistical Analysis

The Graphpad prism 8.0 software was used for graphing and the SPSS 22.0 software was used for data analysis. Student’s *t*-tests or one-way ANOVA was used to analyze the data between groups. Two-way repeated-measures ANOVA was used when analyzing blood pressure at multiple time points. *p* < 0.05 indicates a significant difference. Line and bar plot data are expressed as mean ± SEM.

## Data Availability

Not applicable.

## Supplementary Materials

The following supporting information can be downloaded at: https://www.mdpi.com/article/10.3390/ijms24054955/s1.
