# Supplementary material for: Clostridium butyricum Prevents Dysbiosis and the Rise in Blood Pressure in Spontaneously Hypertensive Rats
Source: Int J Mol Sci. 2023 Mar 4;24(5):4955. doi: 10.3390/ijms24054955 (PMC10002514; doi:10.3390/ijms24054955)
Supplement: Supplementary file 1 [file ijms-24-04955-s001.zip › ijms-2144553-supplementary.pdf]

International Journal of Molecular Sciences

Clostridium butyricum Prevents Dysbiosis and the Rise in Blood Pressure in Spontaneously Hypertensive rats

Xianshu Luo [†], Zhuoyu Han [†], Qing Kong *, Yuming Wang, Haijin Mou, Xuefeng Duan

College of Food Science and Engineering, Ocean University of China, Qingdao, Shandong 266404, China

* Correspondence: kongqing@ouc.edu.cn; Tel.: +86-532-8203-2290;
Fax: +86-532-8203-2272

Table S1 Blood pressure of rats

Group	9	11	13	15
WKY	102±5	104±5	112±2	109±3
WKY-*C. butyricum*	100±2	103±7	107±3	108±5
SHR	148±8	$161\pm3^{\#\#}$	$188\pm7^{\#\#}$	189±7
SHR-*C. butyricum*	146±18	$151\pm7^{\#*}$	$153\pm15^{**}$	$139\pm4^{\#**}$
SHR-butyrate	148±6	$152\pm2^{\#*}$	$145\pm10^{**}$	$144\pm5^{**}$
SHR-CAP	146±4	$147\pm3^{**}$	$144\pm6^{**}$	$134\pm5^{\#**}$

Table S2 Mauchly's test for sphericity

Intrathoracic effect	Mauchly's W	df	Significance	Epsilon[b]		
				GreenhouseGeisser	Huynh-Feldt	Lower limit
Time	0.769	5	0.152	0.840	1	0.333

Table S3 Intrathoracic effect test

Origin		dy	F	Significance
Time	Hypothesis sphericity test	3	37.9	＜0.001
	GreenhouseGeisser	2.521	37.9	
	Huynh-Feldt	3	37.9	
	Lower limit	1	37.9	
Time*Group	Hypothesis sphericity test	15	24.8	＜0.001
	GreenhouseGeisser	12.6	24.8	
	Huynh-Feldt	15	24.8	
	Lower limit	5	24.8	

C. butyricum and butyrate prevented SHR-induced hypertension. Data are shown as the mean ± S.E.M., $^{*}p < 0.05$, $^{**}p < 0.01$ for SHR vs other groups. $^{\#}p < 0.05$, $^{\#\#}p < 0.01$ for each time/group.

Table S4 Primer sets used to detect *C. butyricum* in rat intestine

mRNA targets	Sense	Antisense
My D88	CGGAGGAGATGGGTTTCGAG	CCAGGCATCCAACAAACTGC
NF-κB	GGCATGCGTTTCCGTTACAA	ATTGGGTGCGTCTTAGTGGT
MCT1	GAAAAACTCAAGTCCAAAGAGTCT	TTTCATTGTCTTCTTGGGCTTCT
MCT4	TCAGGAGGCAAGCTGCTGGACGCAA	AGTTGCCCAGCAGCAGCACAAGGGA
GPR43	ATCCTCACGGCCTACATCCT	CAGCAGCAACAACAGCAAGT
GPR41	GCAAGAGAGTGATGGGGCTT	CGGCTTGGAACTTGGAGGAT
β-actin	GACCTTCATTGACCTCAACTACATG	GTCCACCACCGTTGCTGTAGCC
